# A Novel Saccadic Strategy Revealed by Suppression Head Impulse Testing of Patients with Bilateral Vestibular Loss

**DOI:** 10.3389/fneur.2017.00419

**Published:** 2017-08-18

**Authors:** Catherine de Waele, Qiwen Shen, Christophe Magnani, Ian S. Curthoys

**Affiliations:** ^1^CNRS UMR 8257, Cognition and Action Group, Centre Universitaire des Saints-Peres, Universite Paris Descartes, Paris, France; ^2^ENT Department, Salpetriere Hospital, Paris, France; ^3^Vestibular Research Laboratory, School of Psychology, The University of Sydney, Sydney, NSW, Australia

**Keywords:** bilateral vestibular loss, dizziness handicap inventory, horizontal vestibulo-ocular reflex, suppression head impulse test, video head impulse test

## Abstract

**Objective:**

We examined the eye movement response patterns of a group of patients with bilateral vestibular loss (BVL) during suppression head impulse testing. Some showed a new saccadic strategy that may have potential for explaining how patients use saccades to recover from vestibular loss.

**Methods:**

Eight patients with severe BVL [vestibulo-ocular reflex (VOR) gains less than 0.35 and absent otolithic function] were tested. All patients were given the Dizziness Handicap Inventory and questioned about oscillopsia during abrupt head movements. Two paradigms of video head impulse testing of the horizontal VOR were used: (1) the classical head impulse paradigm [called head impulse test (HIMPs)]—fixating an earth-fixed target during the head impulse and (2) the new complementary test paradigm—fixating a head-fixed target during the head impulse (called SHIMPs). The VOR gain of HIMPs was quantified by two algorithms.

**Results:**

During SHIMPs testing, some BVL patients consistently generated an inappropriate covert compensatory saccade during the head impulse that required a corresponding large anti-compensatory saccade at the end of the head impulse in order to obey the instructions to maintain gaze on the head-fixed target. By contrast, other BVL patients did not generate this inappropriate covert saccade and did not exhibit a corresponding anti-compensatory saccade. The latencies of the covert saccade in SHIMPs and HIMPs were similar.

**Conclusion:**

The pattern of covert saccades during SHIMPs appears to be related to the reduction of oscillopsia during abrupt head movements. BVL patients who did not report oscillopsia showed this unusual saccadic pattern, whereas BVL patients who reported oscillopsia did not show this pattern. This inappropriate covert SHIMPs saccade may be an objective indicator of how some patients with vestibular loss have learned to trigger covert saccades during head movements in everyday life.

## Introduction

Bilateral vestibular loss (BVL) patients are often severely handicapped during head movements in their daily life. Profound dysfunction of bilateral semicircular canals usually causes unstable gaze, oscillopsia, and postural imbalance (1). The prevalence of BVL is very low – in the US population it is 28 per 100,000 (2) and the origin is usually difficult to define. Causative factors include ototoxic aminoglycosides, Menière’s disease, Meningitis, systemic autoimmune diseases, Cogan’s syndrome, and positive family history for inner ear diseases, etc (3). In particular, patients often complain of oscillopsia when turning the head rapidly in the horizontal plane, although surprisingly some do not complain of oscillopsia during such rapid head movements.

The conventional video head impulse test (vHIT) [now called head impulse test (HIMP) (4, 5)] quantifies the gain of the vestibulo-ocular reflex (VOR) function (6). In HIMPs, subjects are instructed to maintain gaze on an earth-fixed target during brief, abrupt, unpredictable, horizontal head turns to the left or right. In healthy subjects, the compensatory horizontal slow phase eye velocity matches head velocity, so the gain of the horizontal VOR (HVOR) is around 1.0 (7) and so overt or covert compensatory saccades are only small or are absent (7). By contrast, BVL patients show significantly lower HVOR gain for both horizontal directions and always generate large compensatory covert and/or overt saccades to regain the earth-fixed fixation target (8). In this study, we use the standard terminology (9): a “compensatory” saccade is one which is opposite to the direction of head turn whereas an “anti-compensatory” saccade is one which is in the same direction as the direction of head turn.

**Figure 1:**
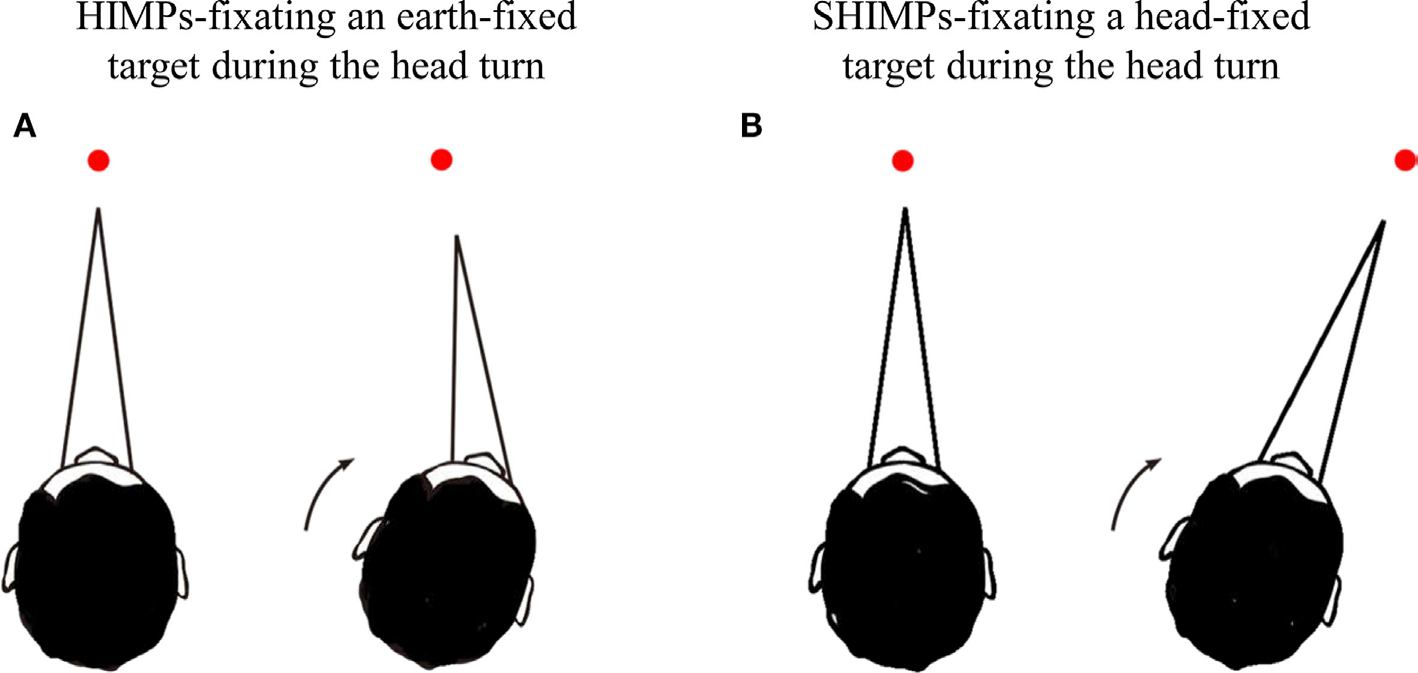
Schematic diagram showing the procedures for head impulse test (HIMP) and SHIMP tests. **(A)** In HIMPs, subjects were instructed to fixate on an earth-fixed laser dot on the wall at 90 cm distance in front of the patient. The clinician applied 20 horizontal head impulses to each side with unpredictable timing and direction. **(B)** In SHIMPs, patients were instructed to fixate a laser spot target projected on the wall at 90 cm distance in front of them from a head-mounted laser. This spot moved with the head and subjects were instructed to stare at the spot. Ten serial impulses were delivered to left and right side, respectively.

Recently, a variant of the HIMPs test has been introduced, called the suppression head impulse paradigm (SHIMPs) (4). It measures VOR and follows the same procedure as HIMPs with one exception: there is no earth-fixed fixation target, instead the patient is instructed to follow the movement of a head-fixed laser spot on the wall during the passive head impulses (see Figure 1). Although slow phase VOR gain is similar in both paradigms, the saccadic performance is very different. The result in SHIMPs is complementary to HIMPs—now healthy subjects generate large anti-compensatory saccades at the end of the head impulse, whereas most patients with BVL usually have very small or absent anti-compensatory saccades. The reason for the anti-compensatory saccade is that in healthy subjects at the onset of the head turn (and for about the first 80 ms) the VOR acts to drive the eyes opposite to head turn and so off the moving target, consequently requiring a large anti-compensatory saccade to regain the target at the end of the head turn (4). The presence and size of this anti-compensatory saccade is an indicator of the level of vestibular function (10). In patients with bilateral VOR deficit, the VOR is minimally functional so the patient’s eyes usually remain on the moving fixation target during the head turn and at the end of the head impulse there is no detectable anti-compensatory saccade. This is in sharp contrast with the performance of the healthy subject (10).

SHIMP testing is now in routine clinical use at Hospital Salpetriere and has been used on many hundreds of patients. In the course of this testing, it has been found that some patients with BVL show, in the SHIMPs paradigm, a saccadic strategy different from the usual strategy described above. Despite the absence of semicircular canal function, some BVL patients consistently generate a compensatory covert saccade during the head turn in the SHIMPs paradigm, even though it is inappropriate because it takes the eyes off target. Thus, these patients must make a large anti-compensatory saccade at the end of the head turn, similar to that produced by healthy subjects, to regain the fixation target. So in these rare cases clinicians cannot rely on the size of the anti-compensatory saccade alone to indicate vestibular loss (10)—they need to inspect the eye movement records and to check the VOR gain as well.

While covert compensatory saccades are well known in patients with unilateral vestibular loss (11–14), their cause has not been established (15). The covert saccades in this group of BVL patients are of special interest, since they cannot be triggered by vestibular input, and the patients appear not to be able to suppress them in accordance with the instructions. By contrast, in the SHIMPs paradigm, patients with unilateral vestibular loss do suppress covert saccades [see Figure 3 of MacDougall et al. (4)].

Interestingly, this new saccadic strategy seems to be related to the subjective experience of oscillopsia of these BVL patients in everyday life—despite their BVL, these patients do not report being troubled by oscillopsia. This novel saccadic strategy is of special interest since it has been suggested that saccades play a major role in recovery after vestibular loss (16–19), and this new SHIMPs paradigm may be a new way of exploring how patients with BVL trigger covert saccades.

## Materials and Methods

### Overview

Eight BVL patients (seven men and one woman; mean age 56 ± 16; min–max: 34–77) with complete bilateral peripheral vestibular deficit were recruited in this study based on the diagnostic criteria by the Classification Committee of the Bárány Society (20). The inclusion criteria were that they demonstrated the following symptoms: postural imbalance, unsteadiness of gait, movement-induced blurred vision (oscillopsia) during walking or most quick head/body movement, and worsening of postural imbalance or unsteadiness of gait in darkness and/or on uneven ground (20). To establish their loss of vestibular function we measured: (1) horizontal semicircular canal function by the video HIMPs, the suppression head impulse paradigm (SHIMPs), the caloric test; (2) otolith function by cervical and ocular vestibular-evoked myogenic potentials (VEMPs) (21, 22); (3) postural stability by the EquiTest, and the Nintendo Wii balance Board (WBB) (23).

Bilateral vestibular loss patients were identified by severe loss of semicircular canal function—they exhibited no responses to caloric testing of both left and right sides with either warm or cold water irrigation function (cold 30°C and warm 44°C water irrigation). Their SPV VOR gain on HIMPs testing was less than 0.35. Both sides were affected about equally—the VOR gain asymmetry between left and right was 2.03 ± 1.33. All eight patients had absent cervical vestibular-evoked myogenic potentials (cVEMPs): no detectable p13-n23 cervical potentials and no detectable ocular vestibular-evoked myogenic potentials (oVEMPs): no n1-p1 ocular potentials were detected on either side in response to short tone bursts of air-conducted sound at 500 Hz at 102 dB SPL (24). All of them fell on condition 5 in Equitest and were unable to maintain balance in the WBB test on foam with eyes closed or on foam at VR0.1 condition (23). We were able to determine the cause of the bilateral loss in four patients (for patients 1,5,7 (Table 1) it was the result of systemic gentamicin; for patient 6 it was genetic).

**Table 1:** Vestibular function test in eight bilateral vestibular loss (BVL) patients.

				Head impulse test (HIMPs)								SHIMPs									
				Vestibulo-ocular reflex (VOR) gain (area)		VOR gain (slope)		Covert saccade		Overt saccade		Peak velocity of anti-compensatory saccade (°/s)		% of anti-compensatory saccades		Latency of anti-compensatory saccade (ms)		Peak velocity of inappropriate saccade (°/s)		Latency of inappropriate saccade (ms)	
												
#	Age	Gender	Dizziness handicap inventory	Left	Right	Left	Right	Left	Right	Left	Right	Left	Right	Left	Right	Left	Right	Left	Right	Left	Right
1	47	M	48	0.03	0.01	0.10	0.01	N	N	Y	Y	31.77	9.37	13	4	N/A	N/A	N/A	N/A	N/A	N/A
2	62	M	56	0.30	0.20	0.19	0.12	Y	Y	Y	Y	64.85	31.63	23	14	N/A	N/A	N/A	N/A	N/A	N/A
3	77	M	12	0.17	0.28	0.17	0.19	Y	Y	N	N	0	66.76	0	30	N/A	N/A	N/A	N/A	N/A	N/A
4	34	F	70	0.32	0.33	0.11	0.10	N	N	Y	Y	0	0	0	0	N/A	N/A	N/A	N/A	N/A	N/A
5	69	M	52	0.21	0.17	0.27	0.22	Y	Y	N	N	178.74	55.79	65	25	300	N/A	205.80	N/A	110	N/A
6	57	M	56	0.09	0.02	0.05	0.00	Y	Y	N	N	199.43	0	75	0	260	N/A	220.29	N/A	130	N/A
7	69	M	28	0.14	0.10	0.14	0.18	Y	Y	Y	Y	193.94	80.89	76	33	290	N/A	203.64	N/A	90	N/A
8	36	M	26	0.27	0.09	0.06	0.05	N	N	Y	Y	170.46	104.92	70	45	240	280	183.36	167.27	130	190

Average latency of anti-compensatory saccade in #5–8 (ms)														270 ± 20							
Average latency of inappropriate saccade in #5–8 (ms)														130 ± 40							

*Patients 1–4 showed low VOR gain (both area and slope) on HIMPs and small SHIMP anti-compensatory saccades. In one side or both sides of patients 5–8, VOR area gain and VOR slope gain remained low, but the appearance of covert saccades led to large SHIMP anti-compensatory saccades. Red highlights: BVL patients who performed covert saccades in SHIMPs*.

All subjects were informed of the vestibular tests and gave written informed consent. The Clinical Research Ethics Committee approved this work, which was registered at ANSM (ID RCB 2014-A00222-45).

### Dizziness Handicap Inventory (DHI) and Complaints of Oscillopsia

In this study, the life quality and complaints of all BVL patients were assessed by the DHI and by an additional question on oscillopsia. The DHI is a self-assessment inventory, including 25 questions to evaluate self-perceived activity limitation and restriction resulting from dizziness (25). To specifically evaluate whether patients complained of oscillopsia, special focus was given to question 11 in the DHI questionnaire “Do quick movements of your head increase your problem?” and we also asked specifically “when you turn rapidly your head horizontally, is your visual scene blurry?”

### Video Head Impulse Test

The function of the horizontal semicircular canals was assessed by using horizontal video-HIT (OtosuiteV^®^, GN Otometrics, Denmark) (6) (Figure 1A). Subjects were instructed to fixate an earth-fixed laser dot on the wall at 90 cm distance in front of them. The clinician applied 20 brief, rapid, horizontal head turns (head impulses) to each side with unpredictable timing and direction. The amplitude of the head rotation was about 18–20°, and the peak head velocity of the impulse was about 180–220°/s, and of the acceleration between 4,500 and 7,500°/s^2^. Eye velocity and head velocity were recorded for each head turn. Two methods of calculating VOR gain from the slow phase eye velocity were used—(1) the area under the desaccaded eye velocity curve divided by the area under the head velocity curve (8). (2) The slope of the function relating eye velocity to head velocity based on a linear regression method as described before (26). The linear regression was calculated in MATLAB R2016a using linear polynomial curve fitting (polyfit) of the eye velocity from the start of the head movement to the peak head velocity, and the slope of this function was used as the second index of VOR gain. Covert saccades were identified as starting before the moment when the head velocity had returned to 0°/s, and overt saccades were identified as the ones starting after the return to 0°/s head velocity within a maximum latency from the start of head rotation of 500 ms. The trials with VOR slope linearity less than 98% and/or overshoot of head velocity of more than 50°/s were excluded from the analysis.

### Suppression Head Impulse Paradigm (SHIMPs)

The SHIMPs testing procedure was exactly the same as for HIMPs with one exception. Participants were instructed to fixate a head-fixed target—a laser spot projected on the wall at 90 cm distance in front of them projected by a head-mounted laser (4) (Figure 1B). This spot moved with the head, and during testing it appeared to subjects that they were looking at a dot which unexpectedly jumped around. Ten impulses were delivered to left and right sides, respectively. To avoid anticipation, the head turn always started from center. Eye velocity and head velocity were recorded in each head rotation.

### Eye Movement Data

An algorithm was developed in MATLAB R2016a (The MathWorks, Inc., USA) to process ASCII data files supplied by ICS impulse (GN Otometrics, Denmark) (10). The algorithm implements saccade detection using a minimal velocity (50–200 /s) and a maximum head-peak to eye-peak duration (600 ms). Only saccades with peak velocities above 200 /s were considered as valid anti-compensatory saccades in our algorithm. The latency of anti-compensatory saccades was defined as the time interval between the onset of the head impulse and the onset of the anti-compensatory saccade response (10).

### Cervical and Ocular VEMPs

Cervical and ocular VEMPs were measured in response to 500 Hz air-conducted sound of 7 ms duration and 1 ms rise time and 102 dB SPL using a Nicolet Viking four apparatus (Nicolet Biomedical Inc., WI, USA) (27). cVEMPs predominantly evaluate the function of sacculo-spinal pathways (28). The function of utriculo-ocular pathways is mainly assessed by oVEMPs (29).

### EquiTest

Equilibrium was evaluated by the Sensory Organization Test in NeuroCom^®^ Balance Manager™ System (NeuroCom^®^ International Inc., USA) (30). Subjects were instructed to stand upright with eyes closed. The support base moved adaptively following the subject’s movement. The function of somatosensory, visual, and vestibular systems was scored according to the change of the body center of pressure.

### Wii Balance Board

Subjects were instructed to maintain balance on the WBB (Nintendo, Japan) with or without a foam rubber mat (Airex AG, Sins, Switzerland, 41 cm × 50 cm × 6 cm) with eyes open and then with eyes closed for 25 s. The moving trajectory of the subject was recorded by a custom app installed in iPod Touch called “VR BalanceRite” (31).

### Statistical Analysis

The average horizontal slow phase eye velocity VOR gain for each side was calculated as the sum of the VOR gains for each trial divided by the number of trials. Average peak anti-compensatory saccade velocity and average peak covert saccade velocity in SHIMPs were calculated as the sum of saccade velocity from the acceptable trials divided by the number of trials. When no anti-compensatory saccade was detected in a particular trial, the peak anti-compensatory saccade velocity was considered as zero.

## Results

### DHI Questionnaire and Complaints of Oscillopsia

The total DHI scores in these patients varied considerably (min–max: 12–70) (Table 1), indicating very different levels of quality of life and compensation of their vestibular deficits between individuals. In particular, some patients complained that they had blurring vision during abrupt horizontal head turns, whereas others did not complain of this. All of our patients had difficulties walking in the dark.

### HIMP and SHIMPs

**Figure 2:**
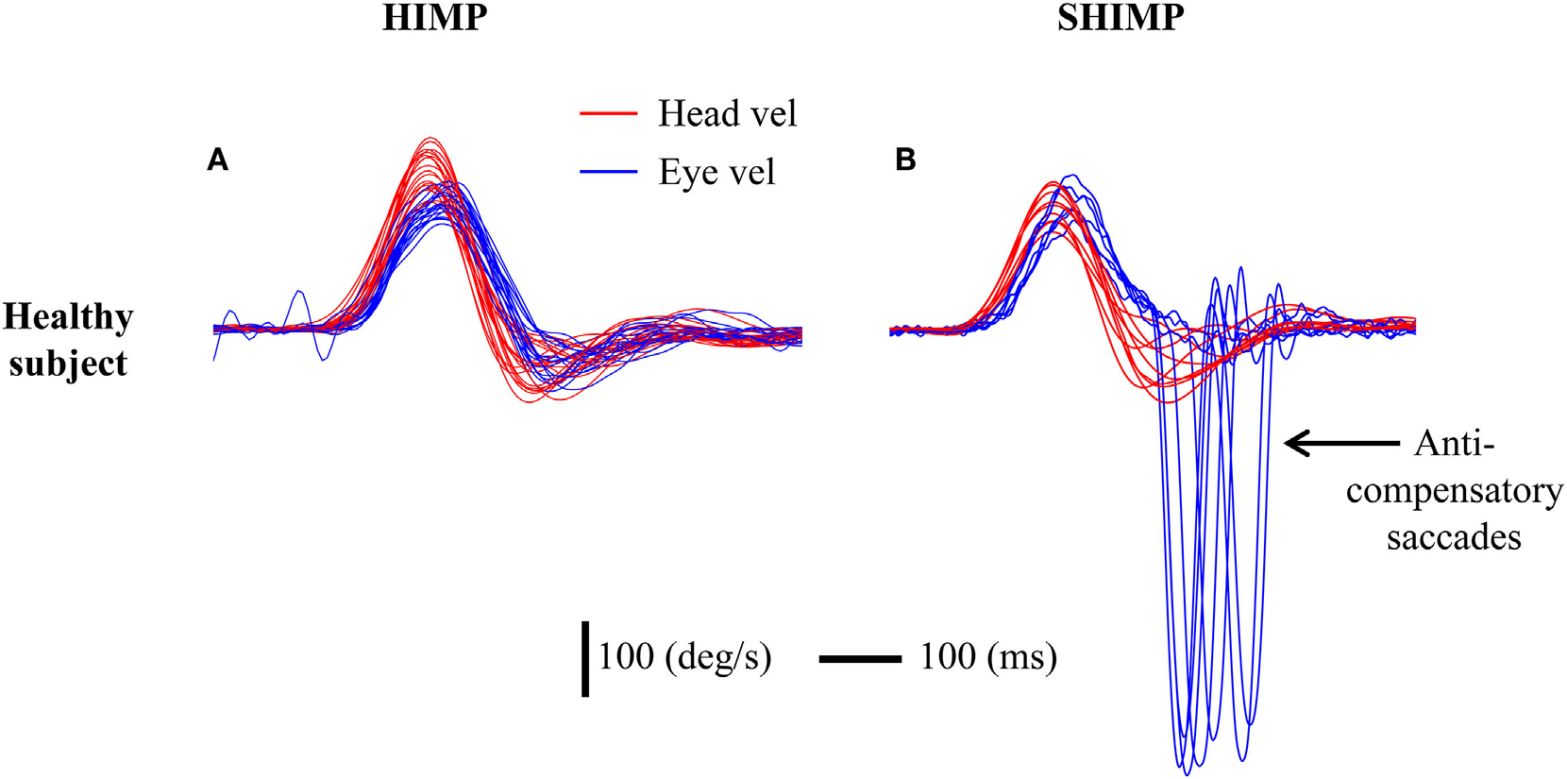
Typical responses in head impulse test (HIMP) and SHIMP procedures in a healthy subject. The figure shows superimposed eye (blue) and head (red) velocity traces for many trials. The subject shows high vestibulo-ocular reflex (VOR) slope gain in both HIMPs and SHIMPs (gains close to 1.0). **(A)** During HIMPs, there are no compensatory saccades. **(B)** In SHIMPs, although the VOR gain was close to 1.0, the subject made no covert saccades but made large anti-compensatory saccades on every trial in order to regain the target. In this and the following figures, the conventions are eye velocity is inverted to facilitate comparison with the head velocity. Red curve: head velocity; blue curve: eye velocity; vertical bar: 100°/s; horizontal bar: 100 ms.

In HIMPs, healthy subjects showed high VOR gain (more than 0.8) and completed the test with only very small compensatory saccades. A typical example of results from a healthy subject [data from our previous study (10)] shows the amplitude of slow phase eye velocity is about equal to that of head velocity (Figure 2A), which means that healthy subjects maintain their gaze very well on the earth-fixed target. By contrast, in SHIMPs, after each rapid head impulse, the healthy subject generated a large anti-compensatory saccade that was in the *same* direction as the head turn, in order to return their gaze to the head-fixed target due to healthy HVOR (Figure 2B). This anti-compensatory saccade was necessitated because, during the head turn, the VOR drove the eyes off the target as explained above.

**Figure 3:**
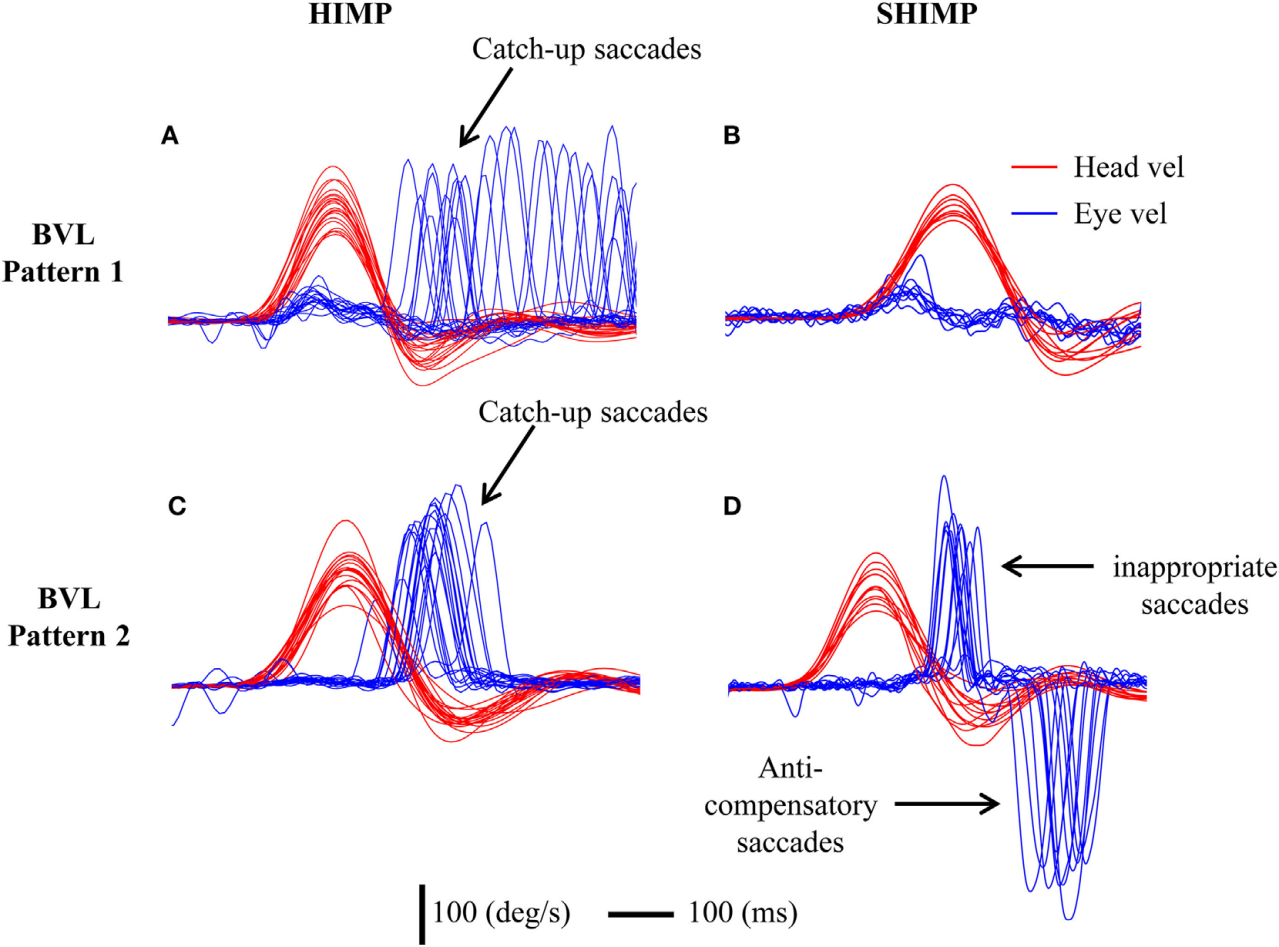
To show the different saccadic patterns in head impulse test (HIMP) and SHIMP procedures in two bilateral vestibular loss (BVL) patients. Eye velocity has been inverted to facilitate comparison with head velocity. Both patients show low vestibulo-ocular reflex (VOR) gains during head impulses. **(A)** A BVL patient with low VOR gain and mainly overt catch-up saccades in HIMPs who **(B)** did not perform any anti-compensatory saccades in SHIMPs. **(C)** A different BVL patient with low HIMP gain and clustered early overt catch-up saccades in HIMPs who **(D)** made covert saccades in SHIMPs followed by large anti-compensatory saccades on every trial because of the covert saccades in SHIMPs.

In HIMPs BVL patients had low VOR area gain (min–max: 0.01–0.33) for both left and right sides (Table 1). The value of the VOR slope gain was also relatively low in BVL patients (min–max: 0–0.27). BVL patients either made only covert saccades (*n* = 3), or only overt saccades (*n* = 3), or a mixture of both covert and overt saccades (*n* = 2). Covert and/or overt catch-up saccades were needed to regain the earth-fixed target after the rapid head turn (Figures 3A,C).

**Figure 4:**
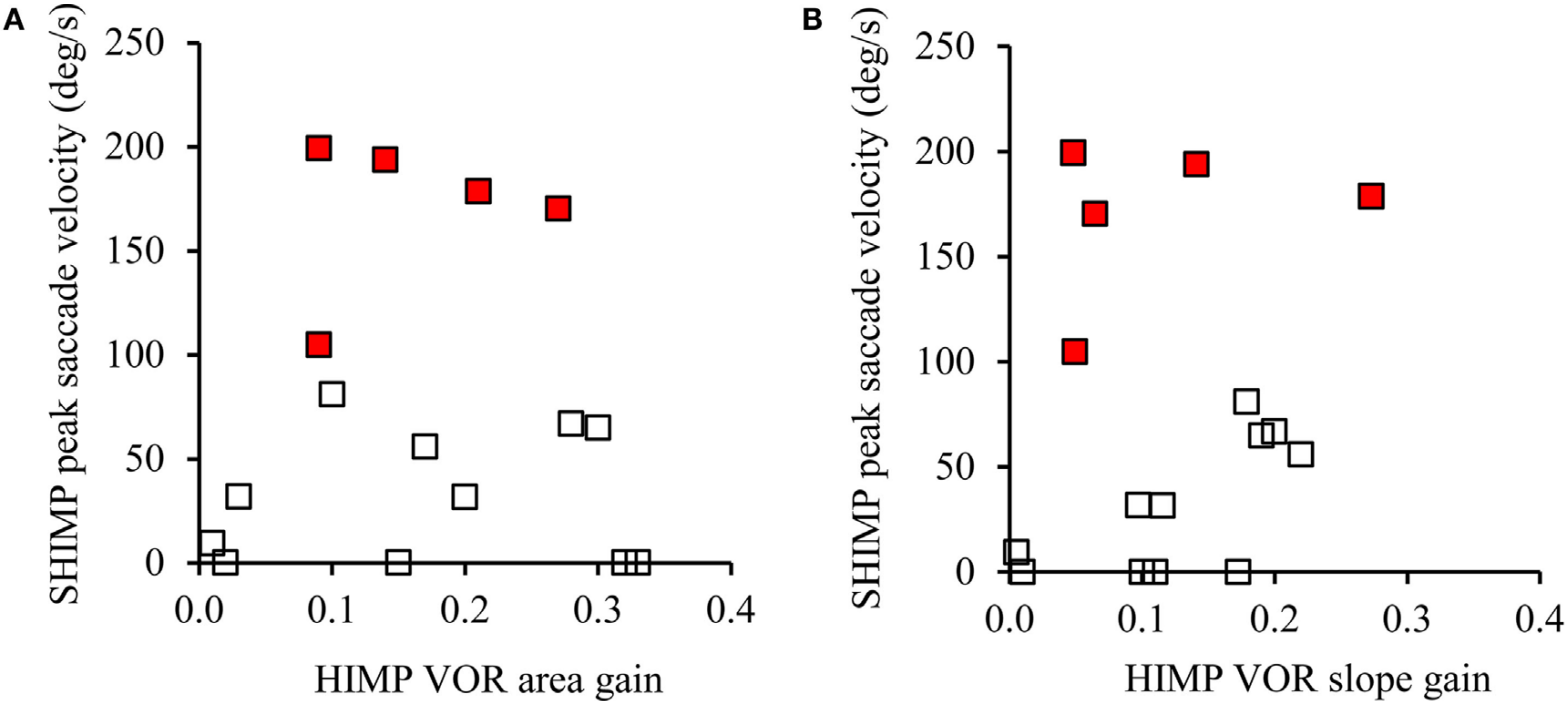
The relation between vestibulo-ocular reflex (VOR) gain and average peak anti-compensatory eye velocity (°/s) in the SHIMPs paradigm as a function of VOR area gain **(A)** and VOR slope gain **(B)** in eight bilateral vestibular loss (BVL) patients (left and right sides). Some patients had low head impulse test (HIMP) gain/HIMP slope and low peak saccade velocity (open squares): these patients performed small or no anti-compensatory saccades in SHIMPs. On the other hand, other BVL patients (red squares) showed low VOR gain by either measure, but had large anti-compensatory saccades in SHIMPs because of earlier covert saccades.

In SHIMPs, the eye movements of most BVL patients followed the head-fixed target during the whole duration of the head turn, from the beginning to the end, because their reduced or absent HVOR did not drive their eyes off the target (Figure 3B). All BVL patients showed low slow phase eye velocity VOR gains in SHIMPs, similar to their VOR gain in HIMPs (Figure 4). Consequently, BVL patients did not usually perform anti-compensatory saccades (Table 1). However, in SHIMPs, some BVL patients consistently made inappropriate covert saccades during the head turn which necessitated large anti-compensatory saccades at the end of the head impulse (Figure 3D).

Covert saccades in the HIMPs paradigm are compensatory for head turn, and act to return gaze to an earth-fixed target and so reduce gaze error at the end of the head impulse. In the SHIMPs paradigm, the covert saccade in BVL patients is also compensatory for head turn, but it acts to *remove* gaze from the head-fixed target, and so acts to *increase* gaze error. That is why we called it “inappropriate.” The covert saccade in the SHIMPs paradigm drives the eyes off the target and so necessitates a large corrective saccade (an anti-compensatory saccade) at the end of the head turn to overcome the large gaze error and return the eyes to the target. Covert saccades in HIMPs and SHIMPs are, thus, totally different—one reduces gaze error, the other increases gaze error.

**Figure 5:**
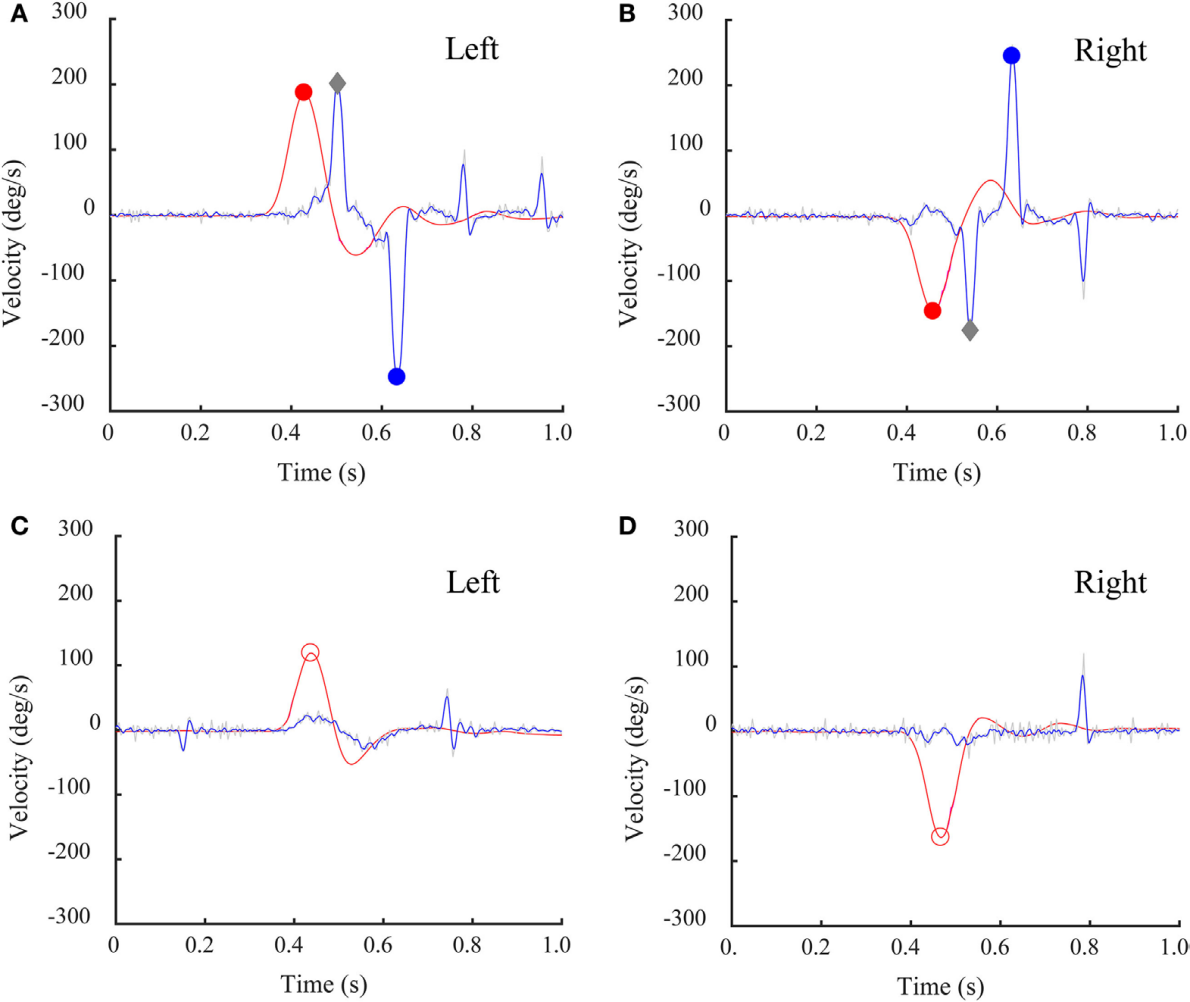
Individual time series records of a bilateral vestibular loss (BVL) patient’s SHIMP results with a covert saccade and corresponding SHIMP anti-compensatory saccades. Eye velocity has been inverted to facilitate comparison with head velocity. **(A,B)** This BVL patient, with low head impulse test (HIMP) gain, made a covert saccade followed by a large anti-compensatory saccade for both left **(A)** and right **(B)** rotation. Gray diamonds: peak saccade velocity of covert saccades. **(C,D)** This BVL patient with low HIMP gain did not make a covert saccade and did not make a corresponding anti-compensatory saccade for head turns to either side. Red circles: peak head velocity; blue circles: peak saccade velocity.

The average latency of the inappropriate covert saccade was 130 ± 40 ms (min–max: 90–190 ms) from the beginning of head turn and in almost every case was followed by a large anti-compensatory saccade. The amplitude of the inappropriate covert saccade ranged from 167 to 220°/s. The amplitude of the corresponding anti-compensatory saccade was from 205 to 382°/s and its average latency was 270 ± 20 ms (min–max: 240–300 ms), which was consistent with the size of anti-compensatory saccades in healthy people published previously (10). This strategy can be seen in detail for head turns to both left side (Figure 5A) and right side (Figure 5B) in some patients. For comparison, sample responses of a patient who did not make inappropriate covert saccades are shown in Figures 5C,D.

## Discussion

By using this new SHIMPs test paradigm, we have been able to show that some BVL patients untroubled by oscillopsia during rapid head movements in everyday life, consistently make inappropriate covert saccades during rapid head movements. The inappropriate covert saccades so clearly revealed by the SHIMPs paradigm suggest that the BVL patients may, almost automatically, generate a compensatory covert saccade during any abrupt head movement. In the SHIMPs protocol the apparently automatic covert saccade is inappropriate—in the usual HIMPs paradigm such a covert saccade would be appropriate because it would be acting to compensate for the head turn to keep the eyes on the target, but in SHIMPs the covert saccade is opposite to the instructed task, because it takes the eyes *off* the target. We have only been able to show this apparently automatic covert saccade by testing a select group of patients with complete BVL in the new SHIMPs paradigm.

What we report here is that during a head impulse these BVL patients make a covert *compensatory* saccade (i.e., a saccade opposite to the direction of head turn), so it is exactly the opposite of the covert *anti-compensatory* eye movement during the head impulse (i.e., a saccade in the direction of head turn) reported by Heuberger et al. (32). (Please see Figure 1 of their paper.) Skilled clinicians carrying out head impulse testing (Dr. de Waele and Dr. Manzari) report that only patients who do not understand the instructions or who are trying to falsify the head impulse test, make the covert anti-compensatory saccades during the head impulse which Heuberger et al. (32) reported and showed in their Figure 1. Eyelid artifact in video recording can also generate an apparent anti-compensatory saccadic eye movement during a head impulse [Figure 8 of Halmagyi et al. (5)].

The fact that some BVL patients made large anti-compensatory saccades at the end of the head impulse on left, right, or both sides in SHIMPs testing raises an issue in diagnosis. If the VOR gain had not been checked, it might be thought that these patients had normal vestibular function, since the presence and amplitude of such anti-compensatory saccades at the end of the head impulse is similar to the response of healthy subjects. However, inspection of the eye velocity record shows that, unlike healthy subjects, the BVL patients had very low VOR gain and the anti-compensatory saccade was preceded by an inappropriate compensatory covert saccade during the head impulse. The presence of this inappropriate covert saccade underscores the importance of the universal instruction for vHIT testing—always look at the eye movement records first. Compared to other BVL patients who made small or no anti-compensatory saccades in SHIMPs, these BVL patients had few complaints of oscillopsia, which indicated a better adaptation in everyday life.

The two methods of calculating VOR gain (area vs slope) were used here since in the SHIMPs paradigm, it is difficult to properly desaccade the eye velocity data and so VOR gain can be in error. However, in this study, across trials and across subjects this was not a factor in these results.

There are only a small number of BVL patients—because patients with severe BVL are not common. Further not all BVL patients showed the inappropriate covert saccades, but the point of this paper is to show the existence of this new consistent saccadic strategy in some BVL patients, and the evidence from four patients shows clearly that the phenomenon exists.

What triggers the inappropriate covert saccades? Covert saccades in HIMPs have a short latency, occurring in the first 200 ms (min–max: 90–190 ms) after the onset of head rotation (14). They might be triggered by neck proprioceptors that are activated during head movement. There is evidence for a cervico-ocular response at around this latency in a human patient with total surgical bilateral loss (33) (Figure 2). However, we cannot eliminate vision as a possible trigger (34). In everyday life, vestibular loss induces discrepancies between head movement and compensatory eye movements and the resulting retinal smear may trigger the covert saccade revealed by the SHIMPs paradigm. Cognitive processing is not likely to be the cause because the covert saccade is so early.

We did not find a direct correlation between the DHI total score and the presence or absence of covert saccades in SHIMPs in our BVL patients. However, four BVL patients with very low VOR gain in both HIMPs and SHIMPs (0–0.33) and inappropriate covert saccades in SHIMPS reported to our specific questions that their visual scene did not become blurry when the head was rapidly turned horizontally. By contrast, four other BVL patients without covert saccades strategy reported that their visual scene *did* become blurry during rapid head turns. Those associations suggest that questions about horizontal oscillopsia may be useful to evaluate the performance of BVL patients.

It had been thought that as the process of vestibular compensation takes place, VOR gain improves and allows recovery of stable retinal images during head movement, reviewed in Ref. (35, 36). Evidence shows that this is not true for head impulses: 1 year after surgical unilateral vestibular loss, the VOR gain of a group of patients was unchanged compared to the VOR gain immediately after their surgical loss (37). Instead saccades would appear to be the vehicle for recovery (38), since the pattern of corrective saccades does change during recovery (16–19). This will affect subjective experience because visual perception is reduced before, during, and after a saccade by a neural process known as saccadic suppression (19, 39, 40). So the visual experience of oscillopsia produced by retinal smear during a head movement due to an inadequate VOR will be reduced. A covert saccade during a head movement would appear to be an effective way of eliminating the subjective experience of oscillopsia, and paradigms training subjects to make covert saccades should be used for rehabilitation of patients with vestibular loss. These suggestions are in accord with the recent evidence showing progressive clustering of saccades in patients recovering from vestibular loss (18). For the reasons above, we suggest the occurrence of such a cluster of covert saccades will be accompanied by a reduction in reports of oscillopsia and improved patient experience. These same covert saccades, while acting to reduce or eliminate retinal smear due to an inadequate VOR will, because of saccadic suppression (19, 39, 40) also reduce the detectability of visual stimuli (such as letters in dynamic visual acuity tests), presented around the time of the covert saccades. Because of this saccadic suppression by covert saccades, measuring dynamic visual acuity during head impulses (41, 42) does not index purely vestibular function.

## Conclusion

SHIMPs are a novel paradigm for studying vestibulo-ocular performance. It gives more precise information on the gain of HVOR compared to HIMPs because, for most patients, the evaluation of the gain is not affected by covert saccades. The subject’s task in SHIMPs is natural and intuitive—the person simply has to follow a moving dot during passive head movements, instead of the rather awkward and unnatural task for the usual HIMPs paradigm of maintaining gaze on an earth-fixed target during passive head movement. The presence of covert saccades is worth further exploration since it would appear to be a rehabilitation strategy, and the anti-compensatory saccade may be an objective indicator of rehabilitation showing how well patients are learning to generate a covert saccade during head movements. A compensatory covert saccade apparently independent of vestibular input is a very useful response for minimizing vestibular loss, and the SHIMP paradigm has laid this strategy bare. It could not be detected with the standard HIMPs paradigm.

## Ethics Statement

All subjects were informed of the vestibular tests and gave written informed consent. The Clinical Research Ethics Committee approved this work, which was registered at ANSM (ID RCB 2014-A00222-45).

## Author Contributions

CW initiated the study, tested the patients, and wrote most of the paper; QS assisted in testing and wrote some of the paper; CM wrote the computer algorithms for acquiring, displaying, and quantifying the data; IC wrote some of the paper. All authors approved the final version.

## Conflict of Interest Statement

IC is an unpaid consultant to GN Otometrics, Taastrup, Denmark, but has received support from GN Otometrics for travel and attendance at conferences and workshops. For all other authors, the research was conducted in the absence of any commercial or financial relationships that could be construed as a potential conflict of interest. The reviewer, SC, and handling editor declared their shared affiliation and the handling editor states that the process nevertheless met the standards of a fair and objective review.

## Abbreviations

BVL, bilateral vestibular loss; DHI, the Dizziness Handicap Inventory; HIMP, conventional head impulse test paradigm; HVOR, horizontal vestibulo-ocular reflex; SHIMP, suppression head impulse paradigm; VEMPs, vestibular-evoked myogenic potentials; vHIT, video head impulse test; VOR, vestibulo-ocular reflex; WBB, the Nintendo Wii Balance Board.
